# Onset of action of the β3-adrenoceptor agonist, mirabegron, in Phase II and III clinical trials in patients with overactive bladder

**DOI:** 10.1007/s00345-014-1244-2

**Published:** 2014-01-24

**Authors:** Christopher R. Chapple, Victor W. Nitti, Vik Khullar, Jean Jacques Wyndaele, Sender Herschorn, Philip van Kerrebroeck, Mary Beth Blauwet, Emad Siddiqui

**Affiliations:** 1 Department of Urology, Royal Hallamshire Hospital, Sheffield Teaching Hospitals, Glossop Road, Sheffield, UK; 2 Department of Urology, NYU Langone Medical Center, New York, NY USA; 3 Urogynaecology Department, St. Mary’s Hospital, Imperial College, London, UK; 4 Department of Urology, University of Antwerp and University Hospital Antwerp, Antwerp, Belgium; 5 Division of Urology, University of Toronto, Toronto, ON Canada; 6 Department of Urology, Maastricht University Medical Center, Maastricht, The Netherlands; 7 Department of Data Science, Biostatistics, Astellas Pharma Global Development, Northbrook, IL USA; 8 Astellas Pharma Europe Ltd, Chertsey, Surrey UK

**Keywords:** Mirabegron, Overactive bladder, β_3_-Adrenoceptor agonist, Onset of action

## Abstract

**Purpose:**

Long-term persistence with pharmacotherapy for overactive bladder (OAB) requires a drug with an early onset of action and good efficacy and tolerability profile. Although antimuscarinics improve OAB symptoms within 1–2 weeks of initiating treatment, adherence after 3 months is relatively poor due to bothersome side effects (e.g., dry mouth and constipation). Mirabegron, a β_3_-adrenoceptor agonist, has demonstrated significant improvements in key symptoms of OAB and good tolerability after 12 weeks in Phase III studies.

**Methods:**

This was a prespecified pooled analysis of three randomized, double-blind, placebo-controlled, 12-week studies, and a Phase II study, to evaluate efficacy and tolerability of mirabegron 25 and 50 mg versus placebo. The main efficacy endpoints were change from baseline to week 1 (Phase II only), week 4, and final visit in mean number of incontinence episodes/24 h, micturitions/24 h, and mean volume voided/micturition (MVV).

**Results:**

A significant benefit for mirabegron 25 and 50 mg versus placebo was evident at the first assessment point, 4 weeks after initiation of therapy, in Phase III studies for incontinence, micturitions, and MVV. The earliest measured benefit was after 1 week, in the Phase II study. Quality-of-life parameters also significantly improved with mirabegron 25 and 50 mg as early as week 4. Significant benefits continued throughout the studies. Mirabegron was well tolerated.

**Conclusions:**

The early onset of action and good overall efficacy and tolerability balance that mirabegron offers may lead to high rates of persistence with mirabegron in the long-term treatment of OAB.

## Introduction

Overactive bladder (OAB) affects 12–16 % of adults across Europe, North America, and Japan [1–4]. Its prevalence increases with age, affecting ~30 % of individuals >65 years. Chronic, bothersome OAB-associated symptoms significantly impact quality of life (QOL) [5, 6] and increase the likelihood of sleep deprivation, depression [7, 8], falls, and fractures [9].

Successful therapy and patient persistence are dependent on early onset of action and low incidence of side effects; therefore, it is important to determine the earliest time at which a therapy shows efficacy and to evaluate adverse events (AEs). Many medications have demonstrated efficacy in reducing OAB symptoms, but efficacy endpoints in OAB studies are evaluated at set timepoints after treatment initiation, with 4 weeks often the earliest assessment.

Oral antimuscarinic drugs, the current mainstay for OAB treatment, are associated with improvements in urgency and frequency as early as 1 week after initiating treatment [10–14]; however, there is heterogeneity among antimuscarinics with respect to onset and duration of action. Furthermore, some patients do not respond adequately to antimuscarinics [15], and/or experience intolerable AEs (e.g., dry mouth, constipation, and blurred vision) due to non-selective anticholinergic effects [16]. Such patients either persist with unsatisfactory treatment or discontinue therapy. Consequently, antimuscarinics are often associated with poor levels of treatment adherence in patients suffering from OAB [17].

Clinicians who treat OAB generally evaluate patients 4 weeks post-initiation of therapy, in order to ascertain where there is inadequate efficacy and the need for dose escalation [18]. Early resolution of symptoms may help improve adherence and avoid use of higher doses of antimuscarinics, with its associated increased risk of AEs.

Mirabegron, a β_3_-adrenoceptor agonist, is approved for OAB treatment in Japan, the USA, Canada, and the European Union and represents the first in a new class of drugs with a mechanism of action distinct from antimuscarinics [19, 20]. Labeling differs between countries, with a recommended starting dose of 25 mg once-daily (QD) in the USA and Canada, with an option to increase to 50 mg QD, and recommended doses of 50 mg QD in Japan and Europe, with the 25 mg dose reserved for special populations (e.g., those with severe renal impairment or moderate hepatic impairment). Efficacy and safety of mirabegron have been investigated in three large 12-week international Phase III studies [21–23]. Significant improvements in key symptoms of OAB: urgency, frequency, and incontinence, were evident after 12 weeks.

## Methods

This was a prespecified pooled analysis of three randomized, double-blind, placebo-controlled, 12-week studies, evaluating efficacy and tolerability of mirabegron 25, 50, and 100 mg (study 178-CL-046 [NCT00689104] [21], study 178-CL-047 [NCT00662909] [22], study 178-CL-074 [NCT00912964]) [23]. Data from a European Phase II study (study 178-CL-044 [NCT00337090]) [20] in which patients received mirabegron 25, 50, 100, and 200 mg were also examined to establish the onset of action of mirabegron. Data for unlicensed doses (100 and 200 mg) are not presented.

All studies were conducted in accordance with ethical principles derived from the Declaration of Helsinki, Good Clinical Practice, and International Conference of Harmonization Guidelines. All patients provided written informed consent.

The studies shared similar designs and enrolled males and females ≥18 years with symptoms of OAB for ≥3 months. Following a 2-week placebo run-in period to establish baseline urinary values and eligibility, patients were randomized if, during a 3-day micturition diary period, they recorded ≥8 micturitions/24 h and ≥3 urgency episodes (based on urgency grade 3 or 4 using Patient Perception of Intensity of Urgency Scale) with/without urgency incontinence. Patients were randomized equally to receive the following oral once-daily treatments for 12 weeks:

- *Study 178-CL-044* placebo, mirabegron 25, 50, 100, 200 mg, tolterodine ER 4 mg
- *Study 178-CL-046* placebo, mirabegron 50, 100 mg, tolterodine ER 4 mg
- *Study 178-CL-047* placebo, mirabegron 50, 100 mg
- *Study 178-CL-074* placebo, mirabegron 25, 50 mg.

Efficacy measures were recorded in a patient micturition diary over 3 days prior to clinic visits: at baseline and week 1 (Phase II study only), weeks 4, 8, 12, and final visit (end of treatment, i.e., last on-treatment assessment including patients not completing week 12 visit).

The main efficacy endpoints in this analysis were change from baseline to week 1 (Phase II only), week 4, and final visit in mean number of incontinence episodes/24 h, micturitions/24 h, and mean volume voided/micturition. Additional efficacy endpoints were changes in mean numbers of urgency episodes (grades 3 or 4)/24 h, urgency incontinence episodes/24 h, mean level of urgency, QOL scores on the International Consultation on Incontinence Questionnaire-Overactive Bladder (ICIQ-OAB) and ICIQ-OABqol for the Phase II study; and change in Overactive Bladder Questionnaire (OAB-q) scores for Phase III studies. Tolerability was assessed according to discontinuation rates and reasons for discontinuation.

The safety analysis set (SAF) comprised all randomized patients who took ≥1 dose of double-blind study drug; the full analysis set (FAS) comprised SAF patients who had ≥1 micturition measurement at baseline and ≥1 post-baseline micturition measurement; the FAS-incontinence (FAS-I) set comprised FAS patients who reported ≥1 incontinence episode at baseline. Efficacy analyses were performed using the FAS except for incontinence episode endpoints, which used the FAS-I. Safety analyses were performed using the SAF.

Analysis of covariance (ANCOVA) was performed on the 178-CL-044 population (with treatment group and country as fixed factors and baseline as a covariate), the pooled population (treatment group, sex, and study as fixed factors and baseline as a covariate) and the 178-CL-074 population (treatment group, sex, and geographical region as fixed factors, baseline as a covariate). For incontinence and urgency incontinence endpoints in the pooled population and 178-CL-074, stratified rank ANCOVA was used for hypothesis testing. All other hypothesis testing was performed using ANCOVA. Based on the ANCOVA, least squares (LS) mean estimates for mean changes from baseline within treatment groups and differences between each mirabegron treatment group and placebo were derived.

## Results

### Patient demographics and baseline characteristics

Patient demographics and baseline characteristics were comparable across studies and treatment groups (Table 1). Most patients were female (~70 % in the Phase III studies, ~90 % in the Phase II study).

**Table 1:** Patient demographics and baseline characteristics

	Study 178-CL-044 (FAS)			Study 178-CL-074 (FAS)		Pooled Phase III studies (FAS)	
	Placebo (*n* = 166)	Mirabegron		Placebo (*n* = 415)	Mirabegron 25 mg (*n* = 410)	Placebo (*n* = 1,328)	Mirabegron 50 mg (*n* = 1,324)
		25 mg (*n* = 167)	50 mg (*n* = 167)				
Gender (*n*, %)							
Male	15 (9.0)	20 (12.0)	18 (10.8)	127 (30.6)	134 (32.7)	362 (27.3)	382 (28.9)
Female	151 (91.0)	147 (88.0)	149 (89.2)	288 (69.4)	276 (67.3)	966 (72.7)	942 (71.1)
Age (years)							
Mean (SD)	57.1 (12.9)	57.2 (12.1)	56.9 (12.5)	58.2 (13.8)	58.8 (12.7)	59.2 (13.2)	59.7 (12.6)
Range	21–80	20–78	26–84	22–85	22–85	20–95	21–91
Race *n* (%)							
White	166 (100)	162 (97.0)	162 (97.0)	372 (89.6)	373 (91.0)	1,227 (92.4)	1,235 (93.3)
Black or African-American	0	2 (1.2)	0	34 (8.2)	31 (7.6)	80 (6.0)	61 (4.6)
Asian	0	1 (0.6)	0	7 (1.7)	5 (1.2)	13 (1.0)	17 (1.3)
Other	0	1 (0.6)	3 (1.8)	2 (0.5)	1 (0.2)	8 (0.6)	11 (0.8)
Missing	0	1 (0.6)	2 (1.2)	0	0	0	
Height (cm), *n* (SD)	164.5 (7.1)	165.2 (7.7)	164.7 (8.2)	166.9 (9.1)	166.8 (9.3)	166.3 (8.9)	166.4 (9.2)
Weight (kg), *n* (SD)	75.1 (14.3)	75.8 (13.2)	72.9 (13.2)	81.0 (18.8)	82.4 (19.0)	80.4 (18.4)^a^	80.3 (18.3)
Type of OAB, *n* (%)							
Urgency incontinence	74 (44.6)	79 (47.3)	67 (40.1)	117 (28.2)	156 (38.0)	442 (33.3)	491 (37.1)
Mixed	52 (31.3)	41 (24.6)	47 (28.1)	137 (33.0)	124 (30.2)	415 (31.3)	412 (31.1)
Frequency	40 (24.1)	47 (28.1)	53 (31.7)	161 (38.8)	130 (31.7)	471 (35.5)	421 (31.8)
Duration of OAB symptoms (months)	*n* = 63^b^	*n* = 63^b^	*n* = 53^b^				
Mean (SD)	54.2 (66.9)	48.0 (35.7)	45.1 (53.7)	91.4 (96.1)	97.4 (115.1)	86.3 (99.1)	85.2 (93.1)
Previous OAB drug, *n* (%)							
Yes	71 (42.8)	82 (49.1)	77 (46.1)	217 (52.3)	219 (53.4)	704 (53.0)	688 (52.0)

^a^Missing weight for 1 (0.1 %) of subjects in the placebo group

^b^If the day and month were missing or the date was completely missing for the start of OAB symptoms, duration of OAB symptoms was not calculated

### Efficacy: Study 178-CL-044

**Fig. 1:**
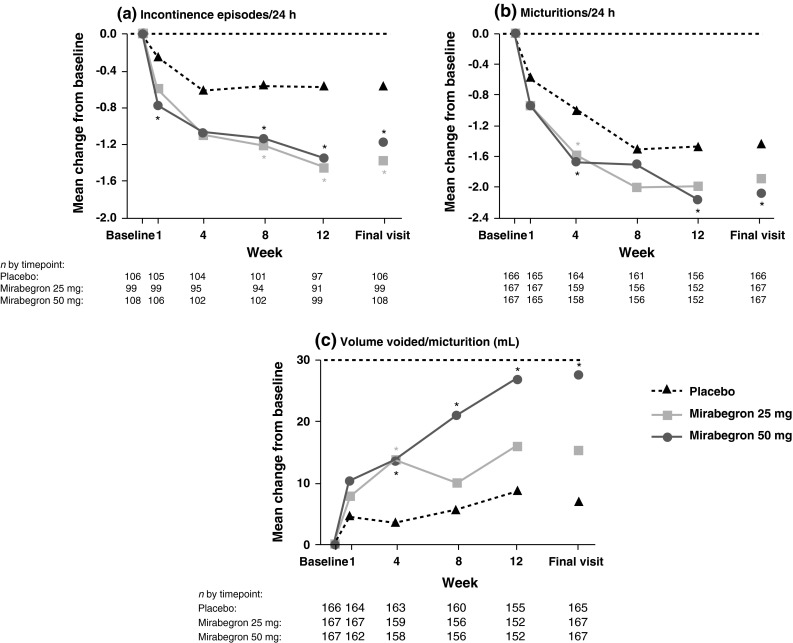
Mean change from baseline at each visit in Study 178-CL-044: **a** the number of incontinence episodes/24 h (full analysis set-incontinence), **b** number of number of micturitions/24 h (full analysis set), and **c** volume voided/micturition (full analysis set). *Statistically significant treatment benefit relative to placebo without multiplicity adjustment (*P* < 0.05). *BL* baseline, *FAS* full analysis set, *FAS-I* full analysis set-incontinence

In this study, which was powered to detect dose response, mirabegron 25 and 50 mg demonstrated improvement over placebo as early as the first measured timepoint of week 1 (Fig. 1). Specifically, there were statistically significant reductions in incontinence episodes/24 h versus placebo for mirabegron 50 mg at week 1. In addition, at week 4, mirabegron 25 and 50 mg were associated with statistically significant reductions versus placebo in micturitions/24 h and volume voided/24 h.

Improvement continued throughout the study, with statistically significant differences versus placebo at final visit for mirabegron 25 and 50 mg for incontinence episodes/24 h, and for mirabegron 50 mg for micturitions/24 h and volume voided/24 h. For the additional endpoints, mirabegron 25 and 50 mg showed improvements versus placebo at all timepoints for urgency episodes (grade 3 or 4)/24 h, urgency incontinence episodes/24 h and level of urgency. These improvements were statistically significant for mirabegron 25 and 50 mg versus placebo at final visit for urgency incontinence episodes/24 h and for mirabegron 25 mg for urgency episodes (grade 3 or 4).

In terms of QOL, mirabegron 25 and 50 mg showed improvements versus placebo at all timepoints for ICIQ-OAB and ICIQ-OABqol (except for 25 mg at week 1).

### Efficacy: study 178-CL-074: 25 mg mirabegron group

Mirabegron 25 mg demonstrated statistically significant reductions from baseline to final visit versus placebo in mean number of incontinence episodes/24 h and micturitions/24 h. Numerical improvements (not statistically significant) were seen at the earliest measured timepoint (week 4) for the key symptoms of OAB (mean number of incontinence episodes/24 h, micturitions/24 h, and mean volume voided/micturition). Improvements continued over the study period. Numerically greater improvements versus placebo were also seen for mirabegron 25 mg at week 4 on the 3 urgency assessments (mean level of urgency, mean number of urgency incontinence episodes/24 h and urgency episodes/24 h [grades 3 or 4]) and on all OAB-q scales and subscales. These improvements were statistically significant versus placebo for the HRQL total score and the Coping and Concern subscale scores.

### Efficacy: pooled analysis: 50 mg mirabegron group

**Fig. 2:**
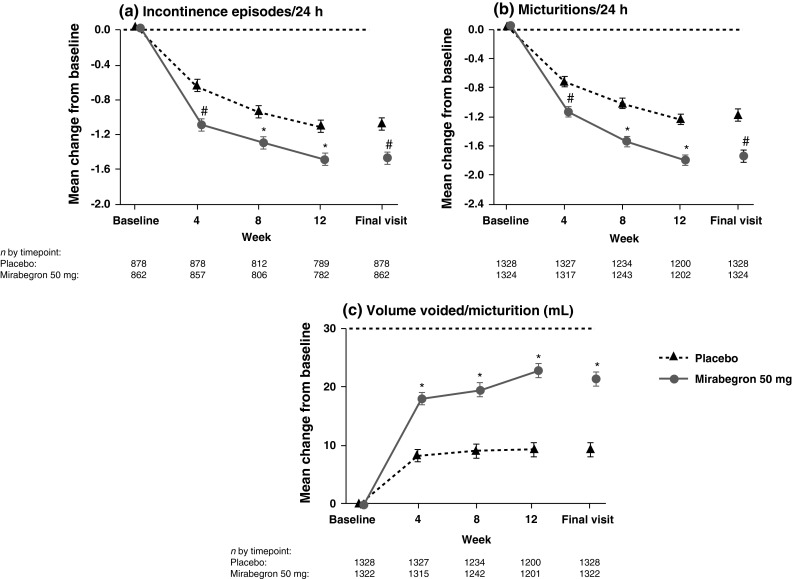
Mean change from baseline (±SE) at each visit in the pooled Phase III studies: **a** the number of incontinence episodes/24 h (full analysis set-incontinence), **b** number of number of micturitions/24 h (full analysis set), and **c** volume voided/micturition (full analysis set). ^#^Statistically significant treatment benefit relative to placebo (*P* < 0.05) with multiplicity adjustment. *Statistically significant treatment benefit relative to placebo (*P* < 0.05) without multiplicity adjustment. *SE* standard error, *FAS* full analysis set

Mirabegron 50 mg demonstrated statistically significantly greater reductions from baseline to earliest measured assessment (week 4) and to final visit versus placebo for incontinence episodes/24 h, micturitions/24 h, and mean volume voided/micturition. Effectiveness was maintained throughout the treatment period (Fig. 2). Statistically significant differences versus placebo were also seen for mirabegron 50 mg at week 4 and final visit in mean number of urgency episodes (grades 3 or 4), urgency incontinence episodes/24 h, OAB-q Symptom Bother, HRQL total, and the Coping and Concern subscales.

### Safety

The overall incidence of TEAEs was similar across treatment groups in all studies (Table 2), and there was no evidence of a dose–response relationship across mirabegron treatment groups for overall rates of TEAEs. The most common TEAEs (in ≥3 % of the total mirabegron group) were headache, hypertension, nasopharyngitis, and urinary tract infection. The most common drug-related TEAEs were hypertension, headache, and dry mouth. The majority of AEs were of mild or moderate severity.

**Table 2:** Overview of treatment-emergent adverse events (SAF)

Number of subjects (%)	Study 178-CL-044 (SAF)			Study 178-CL-074 (SAF)		Pooled Phase III studies (SAF)	
	Placebo	Mirabegron		Placebo (*n* = 433)	Mirabegron 25 mg (*n* = 432)	Placebo (*n* = 1,380)	Mirabegron 50 mg (*n* = 1,375)
	(*n* = 169)	25 mg (*n* = 169)	50 mg (*n* = 169)				
Any TEAE	73 (43.2)	74 (43.8)	74 (43.8)	217 (50.1)	210 (48.6)	658 (47.7)	647 (47.1)
Drug-related TEAE	26 (15.4)	34 (20.1)	38 (22.5)	77 (17.8)	87 (20.1)	232 (16.8)	256 (18.6)
TEAE leading to discontinuation	5 (3.0)	10 (5.9)	3 (1.8)	16 (3.7)	17 (3.9)	46 (3.3)	53 (3.9)
Drug-related TEAE leading to discontinuation	3 (1.8)	8 (4.7)	2 (1.2)	8 (1.8)	11 (2.5)	27 (2.0)	35 (2.5)
SAE	1 (0.6)	1 (0.6)	1 (0.6)	12 (2.8)	7 (1.6)	29 (2.1)	29 (2.1)
Drug-related SAE	NA	NA	NA	2 (0.5)	3 (0.7)	6 (0.4)	7 (0.5)
Common TEAEs by preferred term (reported by ≥3 % in any group)							
Hypertension	0	3 (1.8)	3 (1.8)	37 (8.5)	49 (11.3)	105 (7.6)	103 (7.5)
Nasopharyngitis	12 (7.1)	3 (1.8)	4 (2.4)	14 (3.2)	15 (3.5)	35 (2.5)	54 (3.9)
Urinary tract infection	5 (3.0)	11 (6.5)	3 (1.8)	10 (2.3)	18 (4.2)	25 (1.8)	40 (2.9)
Headache	8 (4.7)	6 (3.6)	8 (4.7)	19 (4.4)	9 (2.1)	42 (3.0)	44 (3.2)
Dry mouth	3 (1.8)	5 (3.0)	3 (1.8)	9 (2.1)	8 (1.9)	29 (2.1)	23 (1.7)
Constipation	2 (1.2)	2 (1.2)	6 (3.6)	5 (1.2)	7 (1.6)	20 (1.4)	22 (1.6)
Influenza	4 (2.4)	5 (3.0)	7 (4.1)	7 (1.6)	3 (0.7)	19 (1.4)	19 (1.4)
Dizziness	1 (0.6)	1 (0.6)	6 (3.6)	2 (0.5)	10 (2.3)	12 (0.9)	13 (0.9)
Asthenia	0	1 (0.6)	6 (3.6)	0	0	2 (0.1)	1 (0.1)
Drug-related* TEAEs by preferred term (reported by ≥3 % in any group)							
Hypertension	0	3 (1.8)	2 (1.2)	23 (5.3)	30 (6.9)	63 (4.6)	65 (4.7)
Headache	4 (2.4)	6 (3.6)	5 (3.0)	9 (2.1)	4 (0.9)	18 (1.3)	28 (2.0)
Dry mouth	3 (1.8)	5 (3.0)	3 (1.8)	8 (1.8)	7 (1.6)	22 (1.6)	13 (0.9)
Dizziness	1 (0.6)	0	6 (3.6)	1 (0.2)	7 (1.6)	8 (0.6)	10 (0.7)

*SAF* safety analysis set, *TEAE* treatment-emergent adverse event, *SAE* serious adverse event, *NA* not available

* Possible or probable, as assessed by the investigator, or records where relationship was missing

## Discussion

OAB is a chronic symptom complex and successful management requires early onset of action of pharmacotherapy plus a good tolerability profile with minimal levels of bothersome side effects in order to encourage long-term persistence with treatment. Although evidence suggests that antimuscarinics improve OAB symptoms within 1–2 weeks of initiating treatment, adherence after 3 months and 1 year is relatively poor due to the often intolerable non-selective anticholinergic effects [17].

These results from a large OAB patient population indicate that mirabegron 25 and 50 mg provide an early onset of action comparable to that demonstrated in previous antimuscarinic studies. A significant benefit was evident at the first assessment after initiation of therapy (4 weeks) in Phase III studies for incontinence, micturitions, and mean volume voided/micturition. However, the earliest measured onset of benefit was at 1 week, as demonstrated in a Phase II study. QOL parameters also significantly improved with mirabegron 25 and 50 mg, as early as week 4, suggesting that these improvements are likely to translate into clinically meaningful benefits. Significant benefits were maintained throughout the treatment period in all studies.

As with most medications, some patients experience symptom improvements rapidly while others require several weeks to experience meaningful improvements. This delay to maximum effect may be because it takes time for patients to modify coping behaviors (e.g., timed voiding, voiding at first sensation of urgency) that they use to control urgency and urge incontinence [24, 25], and it can take several weeks for patients to rely on medication and the relief provided. Clinicians need to inform patients about onset of action to manage expectations regarding time to clinical benefit.

Tolerability is another important determinant of persistence; with mirabegron, this was shown to be comparable with placebo in the pooled Phase III analysis and the Phase II study. In addition, rates of discontinuation due to TEAEs with mirabegron 25 and 50 mg were low and comparable with placebo.

Limitations with the present study relate to the lack of pooled data for the 25 mg dose, with data only available from a single Phase III study. The evaluation of mirabegron at earlier timepoints (e.g., week 1 in the Phase III studies) may also have helped determine whether more immediate improvements are evident.

## Conclusions

Mirabegron at doses of 25 and 50 mg once-daily over 12 weeks demonstrated superior efficacy versus placebo for key symptoms of OAB. The efficacy of mirabegron 50 mg was evident as early as week—the first timepoint measured in the Phase II study—for incontinence episodes, and by week 4—the first measured timepoint in the pooled Phase III studies—for the key OAB symptoms.

Mirabegron possesses the characteristics required to ensure high rates of persistence in the long-term treatment of OAB: an early onset of action and good tolerability profile. Rapid onset of action provides patients with an early perception of benefit from pharmacotherapy and may help patients persist with treatment.
